# *catena*-Poly[[aqua­bis­(3-chloro­benzoato-κ^2^ *O*,*O*′)cadmium]-μ-*N*,*N*-di­ethyl­nico­tin­amide-κ^2^ *N* ^1^:*O*]

**DOI:** 10.1107/S160053681301965X

**Published:** 2013-07-20

**Authors:** Nihat Bozkurt, Tuncay Tunç, Nagihan Çaylak Delibaş, Hacali Necefoğlu, Tuncer Hökelek

**Affiliations:** a Department of Chemistry, Kafkas University, 36100 Kars, Turkey; b Aksaray University, Science Education Department, 68100, Aksaray, Turkey; c Department of Physics, Sakarya University, 54187 Esentepe, Sakarya, Turkey; d Department of Physics, Hacettepe University, 06800 Beytepe, Ankara, Turkey

## Abstract

In the crystal of the title Cd^II^ polymeric complex, [Cd(C_7_H_4_ClO_2_)_2_(C_10_H_14_N_2_O)(H_2_O)]_*n*_, the Cd^II^ cation is chelated by two chloro­benzoate anions and coordinated by two *N*,*N*-di­­ethyl­nicotinamide (DENA) ligands and one water mol­ecule in a distorted NO_6_ penta­gonal–bipyramidal geometry. The Cd^II^ cations are bridged by the pyridine N atom and carbonyl O atom of the DENA ligand to form a polymeric chain running along the *b* axis. Inter­molecular O—H⋯O hydrogen bonds between coordinating water mol­ecules and carboxyl­ate groups link adjacent chains into layers parallel to the *bc* plane. π–π contacts between benzene rings [shortest centroid–centroid distance = 3.912 (2) Å] further stabilizes the crystal structure. In the mol­ecule, weak C—H⋯O hydrogen bonds occur between the pyridine ring and carboxyl­ate groups; the dihedral angles between the carboxyl­ate groups and adjacent benzene rings are 4.6 (3) and 12.8 (3)°, while the benzene rings are oriented at a dihedral angle of 1.89 (13)°.

## Related literature

For niacin, see: Krishnamachari (1974 ▶). For *N*,*N*-di­ethyl­nicotinamide, see: Bigoli *et al.* (1972 ▶). For related structures, see: Çaylak Delibaş *et al.* (2013 ▶); Greenaway *et al.* (1984 ▶); Hökelek *et al.* (1995 ▶); Hökelek & Necefoğlu (1996 ▶); Hökelek *et al.* (2009*a* ▶,*b* ▶,*c* ▶,*d* ▶,*e* ▶,*f* ▶,*g* ▶); Hökelek *et al.* (2011 ▶); Necefoğlu *et al.* (2010*a* ▶,*b* ▶); Sertçelik *et al.* (2013 ▶).
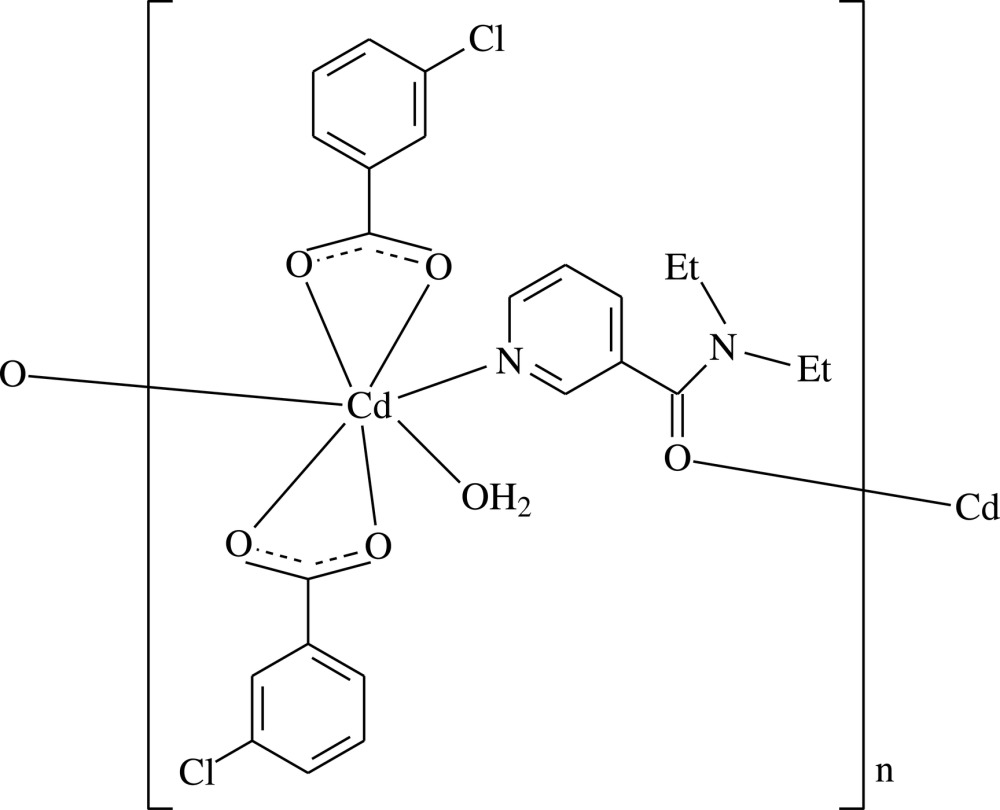


## Experimental

### 

#### Crystal data

[Cd(C_7_H_4_ClO_2_)_2_(C_10_H_14_N_2_O)(H_2_O)]
*M* *_r_* = 619.76
Monoclinic, 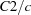

*a* = 25.1809 (5) Å
*b* = 7.0161 (3) Å
*c* = 30.6755 (6) Å
β = 106.203 (3)°
*V* = 5204.2 (3) Å^3^
*Z* = 8
Mo *K*α radiation
μ = 1.09 mm^−1^
*T* = 296 K
0.35 × 0.15 × 0.10 mm

#### Data collection

Bruker SMART BREEZE CCD diffractometer
Absorption correction: multi-scan (*SADABS*; Bruker, 2012 ▶) *T*_min_ = 0.823, *T*_max_ = 0.897
100329 measured reflections
6531 independent reflections
6170 reflections with *I* > 2σ(*I*)
*R*_int_ = 0.026

#### Refinement

*R*[*F* ^2^ > 2σ(*F* ^2^)] = 0.046
*wR*(*F* ^2^) = 0.093
*S* = 1.35
6531 reflections
326 parameters
4 restraints
H atoms treated by a mixture of independent and constrained refinement
Δρ_max_ = 0.77 e Å^−3^
Δρ_min_ = −1.02 e Å^−3^

### 

Data collection: *APEX2* (Bruker, 2012 ▶); cell refinement: *SAINT* (Bruker, 2012 ▶); data reduction: *SAINT*; program(s) used to solve structure: *SHELXS97* (Sheldrick, 2008 ▶); program(s) used to refine structure: *SHELXL97* (Sheldrick, 2008 ▶); molecular graphics: *ORTEP-3 for Windows* (Farrugia, 2012 ▶); software used to prepare material for publication: *WinGX* (Farrugia, 2012 ▶) and *PLATON* (Spek, 2009 ▶).

## Supplementary Material

Crystal structure: contains datablock(s) I, global. DOI: 10.1107/S160053681301965X/xu5721sup1.cif

Structure factors: contains datablock(s) I. DOI: 10.1107/S160053681301965X/xu5721Isup2.hkl

Additional supplementary materials:  crystallographic information; 3D view; checkCIF report

## Figures and Tables

**Table 1:** Selected bond lengths (Å)

Cd1—O1	2.504 (3)
Cd1—O2	2.323 (3)
Cd1—O3	2.421 (3)
Cd1—O4	2.360 (3)
Cd1—O5	2.410 (3)
Cd1—O6	2.314 (3)
Cd1—N1	2.305 (3)

**Table 2:** Hydrogen-bond geometry (Å, °)

*D*—H⋯*A*	*D*—H	H⋯*A*	*D*⋯*A*	*D*—H⋯*A*
O6—H61⋯O2^i^	0.85 (4)	1.94 (4)	2.753 (5)	160 (4)
O6—H62⋯O4^i^	0.86 (4)	2.11 (4)	2.838 (4)	142 (5)
C15—H15⋯O1	0.93	2.52	3.181 (5)	128
C19—H19⋯O3	0.93	2.47	3.130 (5)	128

Symmetry code: (i) 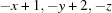.

**Fig. 1.**
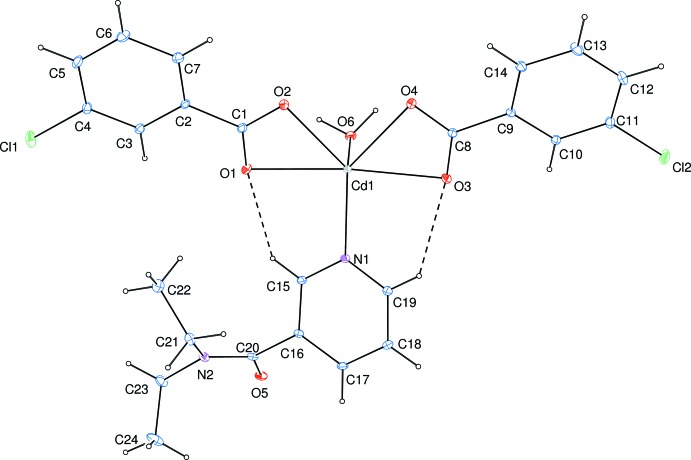
The asymmetric unit of the title molecule with the atom-numbering scheme. Displacement ellipsoids are drawn at the 50% probability level. Hydrogen bonds are shown as dashed lines.

**Fig. 2.**
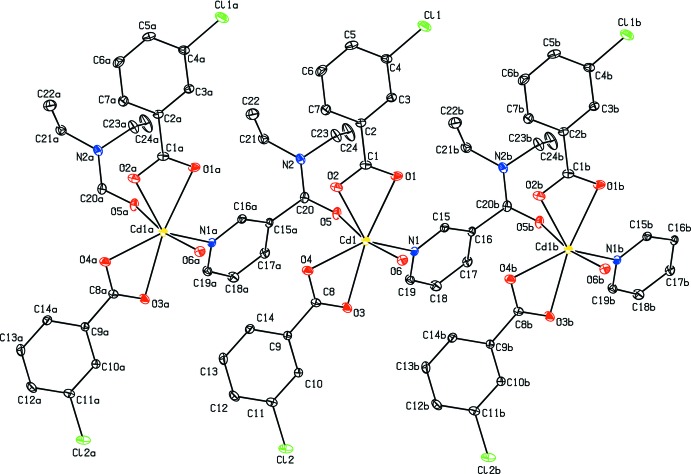
Part of the polymeric chain of the title compound [symmetry codes: (a) x, y - 1, z; (b) x, 1 + y, z]. Hydrogen atoms have been omitted for clarity.

## supplementary crystallographic information

### Comment

As a part of our ongoing investigation on transition metal complexes of nicotinamide (NA), one form of niacin (Krishnamachari, 1974), and/or the nicotinic acid derivative *N*,*N*-diethylnicotinamide (DENA), an important respiratory stimulant (Bigoli *et al.*, 1972), the title compound was synthesized and its crystal structure is reported herein.

The structures of some DENA and/or NA complexes of the Cu^2+^, Zn^2+^ and Co^2+^ ions, [Cu_2_(C_6_H_5_COO)_4_(C_10_H_14_N_2_O)_2_] (Hökelek *et al.*, 1995); [Cu_2_(C_8_H_7_O_2_)_4_(C_6_H_6_N_2_O)_2_] (Necefoğlu *et al.*, 2010*a*); [Zn_2_(C_11_H_14_NO_2_)_4_(C_10_H_14_N_2_O)_2_] (Hökelek *et al.*, 2009*a*); [Zn_2_(C_8_H_8_NO_2_)_4_(C_10_H_14_N_2_O)_2_].2H_2_O (Hökelek *et al.*, 2009*b*); [Zn_2_(C_9_H_10_NO_2_)_4_(C_10_H_14_N_2_O)_2_] (Hökelek *et al.*, 2009*c*); [Zn_2_(C_8_H_7_O_2_)_4_(C_10_H_14_N_2_O)_2_] (Necefoğlu *et al.*, 2010*b*) and [Co_2_(C_11_H_14_NO_2_)_4_(C_10_H_14_N_2_O)_2_] (Hökelek *et al.*, 2011) have also been determined. In these structures, the benzoate ion acts as a bidentate ligand.

The asymmetric unit of the title Cd^II^ complex, [Cd(CB)_2_(DENA)(H_2_O)]_n_, contains two 3-chlorobenzoate (CB), one *N,N*-diethylnicotinamide (DENA) ligands and one coordinated water molecule; the CB ions act as bidentate ligands (Fig. 1). The coordination number of the Cd^II^ ion is six. Intramolecular C—H···O hydrogen bonds (Fig. 1 and Table 2) link the DENA ligand to the carboxyl groups. The O1—Cd1—O2 and O3—Cd1—O4 angles are 53.75 (10)° and 54.23 (9) °, respectively. The corresponding O—M—O (where *M* is a , metal) angles are 53.50 (14)° in [Cu_2_(C_8_H_5_O_3_)_4_(C_6_H_6_N_2_O)_4_] (Sertçelik *et al.*, 2013), 53.45 (4)° and 51.97 (4)° in [Cd(C_7_H_5_O_3_)_2_(C_6_H_6_NO)(H_2_O)_2_].2(H_2_O) (Çaylak Delibaş *et al.*, 2013), 52.91 (4)° and 53.96 (4)° in [Cd(C_8_H_5_O_3_)_2_(C_6_H_6_N_2_O)_2_(H_2_O)].H_2_O (Hökelek *et al.*, 2009*d*), 60.70 (4)° in [Co(C_9_H_10_NO_2_)_2_(C_6_H_6_N_2_O)(H_2_O)_2_] (Hökelek *et al.*, 2009*e*), 58.45 (9)° in [Mn(C_9_H_10_NO_2_)_2_- (C_6_H_6_N_2_O)(H_2_O)_2_] (Hökelek *et al.*, 2009*f*), 60.03 (6)° in [Zn(C_8_H_8_NO_2_)_2_(C_6_H_6_N_2_O)_2_].H_2_O (Hökelek *et al.*, 2009*g*), 58.3 (3)° in [Zn_2_(C_10_H_14_N_2_O)_2_(C_7_H_5_O_3_)_4_].2H_2_O (Hökelek & Necefoğlu, 1996) and 55.2 (1)° in [Cu(Asp)_2_(py)_2_] (where Asp is acetylsalicylate and py is pyridine) (Greenaway *et al.*, 1984). The dihedral angles between the planar carboxylate groups [(O1/O2/C1) and (O3/O4/C8)] and the adjacent benzene rings A (C2—C7) and B (C9—C14) are 4.56 (28)° and 12.84 (26)°, respectively, while that between rings A, B and C (N1/C15–C19) are A/B = 1.89 (13), A/C = 34.83 (13) and B/C = 35.13 (11) °.

In the crystal, the Cd^II^ ions [Cd1···Cd1a = 7.0161 (5) Å; symmetry code: (a) *x*, *y* - 1, *z*] are bridged by the N and O atoms of the DENA ligands forming polymeric chains running along the *b*-axis direction, where the coordination number of each Cd^II^ atom is seven within a CdO_6_N donor set (Fig. 2). The average Cd—O distance is 2.389 (3) Å (Table 1). The Cd atom lies 0.0972 (3) Å below and 0.0127 (3) Å above of the carboxylate groups [(O1/O2/C1) and (O3/O4/C8)], respectively. Strong intermolecular O—H···O hydrogen bonds (Table 2) between water molecules and carboxylate groups link the adjacent chains into layers parallel to the *bc* plane. π···π contacts between the benzene rings Cg1—Cg2^i^, [symmetry code: (i) 1 - *x*, 2 - *y*, - *z*, where Cg1 and Cg2 are the centroids of the rings A (C2—C7) and B (C9—C14), respectively] may further stabilize the structure, with centroid-centroid distance of 3.912 (2) Å.

### Experimental

The title compound was prepared by the reaction of CdSO_4_.8H_2_O (1.283 g, 5 mmol) in H_2_O (100 ml) and diethylnicotinamide (1.780 g, 10 mmol) in H_2_O (10 ml) with sodium 3-chlorobenzoate (1.790 g, 10 mmol) in H_2_O (100 ml). The mixture was filtered and set aside to crystallize at ambient temperature for ten days, giving colorless single crystals.

### Refinement

Atoms H61 and H62 (for H_2_O) were located in a difference Fourier map and were refined freely. The C-bound H-atoms were positioned geometrically with C—H = 0.93, 0.97 and 0.96 Å, for aromatic, methylene and methyl H-atoms, respectively, and constrained to ride on their parent atoms, with *U*_iso_(H) = k × *U*_eq_(C), where k = 1.5 for methyl H-atoms and k = 1.2 for all other H-atoms.

### Crystal data

**Table:**

[Cd(C_7_H_4_ClO_2_)_2_(C_10_H_14_N_2_O)(H_2_O)]	*F*(000) = 2496
*M**_r_* = 619.76	*D*_x_ = 1.582 Mg m^−^^3^
Monoclinic, *C*2/*c*	Mo *K*α radiation, λ = 0.71073 Å
Hall symbol: -C2yc	Cell parameters from 9713 reflections
*a* = 25.1809 (5) Å	θ = 2.5–28.4°
*b* = 7.0161 (3) Å	µ = 1.09 mm^−^^1^
*c* = 30.6755 (6) Å	*T* = 296 K
β = 106.203 (3)°	Rod-shaped, colourless
*V* = 5204.2 (3) Å^3^	0.35 × 0.15 × 0.10 mm
*Z* = 8	

### Data collection

**Table:**

Bruker SMART BREEZE CCD diffractometer	6531 independent reflections
Radiation source: fine-focus sealed tube	6170 reflections with *I* > 2σ(*I*)
Graphite monochromator	*R*_int_ = 0.026
φ and ω scans	θ_max_ = 28.4°, θ_min_ = 1.4°
Absorption correction: multi-scan (*SADABS*; Bruker, 2012)	*h* = −33→33
*T*_min_ = 0.823, *T*_max_ = 0.897	*k* = −9→9
100329 measured reflections	*l* = −41→40

### Refinement

**Table:**

Refinement on *F*^2^	Primary atom site location: structure-invariant direct methods
Least-squares matrix: full	Secondary atom site location: difference Fourier map
*R*[*F*^2^ > 2σ(*F*^2^)] = 0.046	Hydrogen site location: inferred from neighbouring sites
*wR*(*F*^2^) = 0.093	H atoms treated by a mixture of independent and constrained refinement
*S* = 1.35	*w* = 1/[σ^2^(*F*_o_^2^) + (0.0097*P*)^2^ + 22.0143*P*] where *P* = (*F*_o_^2^ + 2*F*_c_^2^)/3
6531 reflections	(Δ/σ)_max_ = 0.001
326 parameters	Δρ_max_ = 0.77 e Å^−^^3^
4 restraints	Δρ_min_ = −1.02 e Å^−^^3^

### Special details

**Table:**

Geometry. All esds (except the esd in the dihedral angle between two l.s. planes) are estimated using the full covariance matrix. The cell esds are taken into account individually in the estimation of esds in distances, angles and torsion angles; correlations between esds in cell parameters are only used when they are defined by crystal symmetry. An approximate (isotropic) treatment of cell esds is used for estimating esds involving l.s. planes.
Refinement. Refinement of F^2^ against ALL reflections. The weighted R-factor wR and goodness of fit S are based on F^2^, conventional R-factors R are based on F, with F set to zero for negative F^2^. The threshold expression of F^2^ > 2sigma(F^2^) is used only for calculating R-factors(gt) etc. and is not relevant to the choice of reflections for refinement. R-factors based on F^2^ are statistically about twice as large as those based on F, and R- factors based on ALL data will be even larger.

### Fractional atomic coordinates and isotropic or equivalent isotropic displacement parameters (Å^2^)

**Table:**

	*x*	*y*	*z*	*U*_iso_*/*U*_eq_	
Cd1	0.430006 (10)	0.86384 (4)	0.032753 (9)	0.03649 (8)	
Cl1	0.67865 (6)	0.6435 (2)	0.24132 (4)	0.0797 (4)	
Cl2	0.20678 (5)	1.2006 (2)	−0.17853 (4)	0.0741 (4)	
O1	0.50773 (11)	0.7328 (5)	0.09538 (10)	0.0534 (7)	
O2	0.50591 (11)	1.0331 (5)	0.07578 (9)	0.0533 (7)	
O3	0.35198 (12)	0.9354 (4)	−0.03179 (10)	0.0547 (8)	
O4	0.41552 (11)	1.1524 (4)	−0.00840 (9)	0.0500 (7)	
O5	0.39003 (12)	0.9782 (4)	0.09065 (10)	0.0498 (7)	
O6	0.47410 (12)	0.7107 (5)	−0.01422 (11)	0.0485 (7)	
H61	0.472 (2)	0.785 (7)	−0.0367 (12)	0.083 (19)*	
H62	0.5081 (12)	0.709 (10)	0.0016 (18)	0.13 (3)*	
N1	0.38036 (12)	0.6005 (4)	0.04343 (10)	0.0349 (6)	
N2	0.42885 (14)	1.1630 (5)	0.15138 (11)	0.0453 (8)	
C1	0.52769 (14)	0.8941 (6)	0.10063 (12)	0.0420 (9)	
C2	0.57856 (13)	0.9332 (6)	0.13935 (12)	0.0368 (8)	
C3	0.60309 (14)	0.7865 (6)	0.16792 (12)	0.0410 (8)	
H3	0.5892	0.6631	0.1627	0.049*	
C4	0.64831 (15)	0.8243 (6)	0.20430 (13)	0.0451 (9)	
C5	0.66979 (17)	1.0049 (8)	0.21233 (15)	0.0575 (12)	
H5	0.7004	1.0287	0.2369	0.069*	
C6	0.6455 (2)	1.1492 (8)	0.18368 (16)	0.0648 (13)	
H6	0.6597	1.2721	0.1888	0.078*	
C7	0.59975 (17)	1.1136 (6)	0.14707 (15)	0.0516 (10)	
H7	0.5835	1.2125	0.1278	0.062*	
C8	0.37094 (14)	1.0967 (5)	−0.03489 (11)	0.0367 (8)	
C9	0.33859 (14)	1.2296 (5)	−0.07074 (11)	0.0328 (7)	
C10	0.29377 (14)	1.1625 (5)	−0.10465 (11)	0.0364 (7)	
H10	0.2847	1.0337	−0.1060	0.044*	
C11	0.26293 (16)	1.2868 (6)	−0.13623 (13)	0.0458 (9)	
C12	0.2747 (2)	1.4770 (7)	−0.13478 (15)	0.0583 (12)	
H12	0.2530	1.5599	−0.1561	0.070*	
C13	0.3196 (2)	1.5445 (6)	−0.10090 (16)	0.0606 (12)	
H13	0.3280	1.6737	−0.0994	0.073*	
C14	0.35155 (18)	1.4214 (6)	−0.06956 (13)	0.0465 (9)	
H14	0.3821	1.4673	−0.0474	0.056*	
C15	0.40358 (14)	0.4560 (5)	0.07000 (12)	0.0363 (7)	
H15	0.4418	0.4552	0.0822	0.044*	
C16	0.37313 (15)	0.3070 (5)	0.08017 (12)	0.0346 (7)	
C17	0.31657 (15)	0.3072 (6)	0.06121 (13)	0.0442 (9)	
H17	0.2951	0.2065	0.0665	0.053*	
C18	0.29234 (16)	0.4580 (7)	0.03445 (15)	0.0523 (10)	
H18	0.2542	0.4629	0.0220	0.063*	
C19	0.32558 (15)	0.6014 (6)	0.02650 (13)	0.0442 (9)	
H19	0.3091	0.7037	0.0085	0.053*	
C20	0.39878 (15)	1.1380 (5)	0.10824 (13)	0.0408 (8)	
C21	0.43608 (18)	1.3440 (7)	0.17587 (14)	0.0530 (10)	
H21A	0.4277	1.3260	0.2046	0.064*	
H21B	0.4101	1.4362	0.1583	0.064*	
C22	0.4944 (2)	1.4228 (8)	0.18486 (17)	0.0724 (14)	
H22A	0.4972	1.5408	0.2012	0.109*	
H22B	0.5026	1.4446	0.1565	0.109*	
H22C	0.5203	1.3328	0.2026	0.109*	
C23	0.4519 (2)	0.9923 (7)	0.17814 (16)	0.0606 (12)	
H23A	0.4863	1.0261	0.2004	0.073*	
H23B	0.4601	0.8969	0.1581	0.073*	
C24	0.4129 (3)	0.9093 (9)	0.2021 (2)	0.097 (2)	
H24A	0.4306	0.8052	0.2210	0.146*	
H24B	0.3802	0.8641	0.1802	0.146*	
H24C	0.4029	1.0056	0.2207	0.146*	

### Atomic displacement parameters (Å^2^)

**Table:**

	*U*^11^	*U*^22^	*U*^33^	*U*^12^	*U*^13^	*U*^23^
Cd1	0.03456 (13)	0.03444 (13)	0.03743 (13)	−0.00848 (11)	0.00502 (9)	0.00642 (11)
Cl1	0.0728 (8)	0.0926 (10)	0.0612 (7)	0.0290 (8)	−0.0022 (6)	0.0143 (7)
Cl2	0.0523 (6)	0.1018 (11)	0.0533 (6)	−0.0002 (7)	−0.0100 (5)	0.0020 (7)
O1	0.0415 (15)	0.0632 (19)	0.0483 (16)	−0.0173 (14)	0.0008 (12)	−0.0009 (14)
O2	0.0414 (15)	0.070 (2)	0.0435 (15)	−0.0062 (14)	0.0043 (12)	0.0154 (15)
O3	0.0528 (16)	0.0446 (16)	0.0570 (17)	−0.0091 (13)	−0.0005 (13)	0.0198 (14)
O4	0.0392 (14)	0.0561 (17)	0.0474 (15)	−0.0072 (13)	−0.0002 (11)	0.0094 (14)
O5	0.0609 (17)	0.0322 (14)	0.0653 (18)	−0.0061 (13)	0.0322 (15)	−0.0015 (13)
O6	0.0440 (16)	0.0526 (17)	0.0512 (17)	−0.0086 (13)	0.0173 (13)	0.0004 (14)
N1	0.0369 (14)	0.0292 (15)	0.0384 (15)	−0.0081 (12)	0.0103 (12)	0.0011 (12)
N2	0.0537 (19)	0.0402 (18)	0.0457 (18)	0.0070 (15)	0.0200 (15)	0.0106 (14)
C1	0.0291 (16)	0.063 (3)	0.0349 (18)	−0.0060 (17)	0.0107 (14)	0.0010 (18)
C2	0.0260 (15)	0.049 (2)	0.0356 (17)	−0.0057 (14)	0.0095 (13)	−0.0025 (15)
C3	0.0344 (17)	0.048 (2)	0.0401 (19)	−0.0016 (16)	0.0102 (15)	−0.0038 (17)
C4	0.0351 (18)	0.060 (3)	0.0383 (19)	0.0069 (17)	0.0074 (15)	−0.0016 (18)
C5	0.041 (2)	0.080 (3)	0.045 (2)	−0.013 (2)	0.0015 (18)	−0.014 (2)
C6	0.062 (3)	0.059 (3)	0.068 (3)	−0.023 (2)	0.008 (2)	−0.014 (2)
C7	0.049 (2)	0.046 (2)	0.058 (2)	−0.0066 (19)	0.0110 (19)	0.002 (2)
C8	0.0339 (17)	0.045 (2)	0.0324 (17)	−0.0001 (15)	0.0108 (13)	0.0058 (15)
C9	0.0377 (17)	0.0326 (17)	0.0300 (16)	−0.0011 (14)	0.0128 (13)	0.0032 (13)
C10	0.0373 (17)	0.0345 (18)	0.0376 (18)	−0.0006 (14)	0.0109 (14)	0.0006 (14)
C11	0.041 (2)	0.060 (3)	0.0341 (19)	0.0080 (18)	0.0072 (15)	0.0051 (18)
C12	0.070 (3)	0.053 (3)	0.048 (2)	0.018 (2)	0.011 (2)	0.018 (2)
C13	0.085 (3)	0.033 (2)	0.061 (3)	0.003 (2)	0.016 (2)	0.007 (2)
C14	0.059 (2)	0.037 (2)	0.040 (2)	−0.0086 (18)	0.0097 (17)	−0.0011 (16)
C15	0.0350 (17)	0.0350 (18)	0.0388 (18)	−0.0089 (14)	0.0102 (14)	0.0001 (15)
C16	0.0413 (18)	0.0294 (16)	0.0366 (17)	−0.0043 (14)	0.0168 (14)	−0.0004 (14)
C17	0.0406 (19)	0.041 (2)	0.054 (2)	−0.0147 (16)	0.0187 (17)	0.0038 (17)
C18	0.0332 (18)	0.060 (3)	0.061 (3)	−0.0097 (18)	0.0097 (17)	0.012 (2)
C19	0.0403 (19)	0.039 (2)	0.052 (2)	−0.0046 (16)	0.0100 (16)	0.0093 (17)
C20	0.0464 (19)	0.0345 (18)	0.049 (2)	−0.0065 (16)	0.0265 (17)	0.0047 (16)
C21	0.061 (3)	0.055 (3)	0.043 (2)	0.009 (2)	0.0142 (19)	0.0029 (19)
C22	0.070 (3)	0.079 (4)	0.062 (3)	−0.010 (3)	0.010 (2)	0.000 (3)
C23	0.069 (3)	0.056 (3)	0.065 (3)	0.022 (2)	0.032 (2)	0.024 (2)
C24	0.116 (5)	0.085 (4)	0.117 (5)	0.037 (4)	0.076 (4)	0.059 (4)

### Geometric parameters (Å, º)

**Table:**

Cd1—O1	2.504 (3)	C9—C10	1.386 (5)
Cd1—O2	2.323 (3)	C9—C14	1.383 (5)
Cd1—O3	2.421 (3)	C10—C11	1.372 (5)
Cd1—O4	2.360 (3)	C10—H10	0.9300
Cd1—O5	2.410 (3)	C11—C12	1.365 (6)
Cd1—O6	2.314 (3)	C12—C13	1.388 (7)
Cd1—N1	2.305 (3)	C12—H12	0.9300
Cd1—C1	2.752 (4)	C13—H13	0.9300
Cd1—C8	2.732 (3)	C14—C13	1.373 (6)
Cl1—C4	1.732 (4)	C14—H14	0.9300
Cl2—C11	1.739 (4)	C15—C16	1.383 (5)
O1—C1	1.230 (5)	C15—H15	0.9300
O3—C8	1.242 (4)	C16—C17	1.380 (5)
O4—C8	1.251 (4)	C16—C20^i^	1.502 (5)
O6—H61	0.856 (17)	C17—C18	1.373 (6)
O6—H62	0.86 (2)	C17—H17	0.9300
O5—C20	1.237 (5)	C18—H18	0.9300
N1—C15	1.330 (4)	C19—C18	1.374 (5)
N1—C19	1.331 (5)	C19—H19	0.9300
N2—C21	1.461 (5)	C20—N2	1.340 (5)
N2—C23	1.476 (5)	C20—C16^ii^	1.502 (5)
C1—O2	1.265 (5)	C21—C22	1.521 (6)
C2—C1	1.509 (5)	C21—H21A	0.9700
C2—C3	1.381 (5)	C21—H21B	0.9700
C2—C7	1.368 (6)	C22—H22A	0.9600
C3—C4	1.380 (5)	C22—H22B	0.9600
C3—H3	0.9300	C22—H22C	0.9600
C4—C5	1.372 (6)	C23—C24	1.500 (6)
C5—C6	1.368 (7)	C23—H23A	0.9700
C5—H5	0.9300	C23—H23B	0.9700
C6—H6	0.9300	C24—H24A	0.9600
C7—C6	1.389 (6)	C24—H24B	0.9600
C7—H7	0.9300	C24—H24C	0.9600
C9—C8	1.497 (5)		
			
O1—Cd1—C1	26.54 (11)	C7—C6—H6	119.8
O1—Cd1—C8	161.03 (10)	C2—C7—C6	120.1 (4)
O2—Cd1—O1	53.75 (10)	C2—C7—H7	119.9
O2—Cd1—O3	135.22 (10)	C6—C7—H7	119.9
O2—Cd1—O4	81.10 (10)	O3—C8—Cd1	62.38 (19)
O2—Cd1—O5	81.83 (10)	O3—C8—O4	122.0 (3)
O2—Cd1—C1	27.21 (11)	O3—C8—C9	118.8 (3)
O2—Cd1—C8	108.24 (11)	O4—C8—Cd1	59.57 (19)
O3—Cd1—O1	170.39 (10)	O4—C8—C9	119.2 (3)
O3—Cd1—C1	162.33 (11)	C9—C8—Cd1	178.0 (3)
O3—Cd1—C8	27.04 (10)	C10—C9—C8	120.1 (3)
O4—Cd1—O1	134.15 (9)	C14—C9—C8	120.7 (3)
O4—Cd1—O3	54.23 (9)	C14—C9—C10	119.1 (3)
O4—Cd1—O5	94.28 (10)	C9—C10—H10	120.1
O4—Cd1—C1	108.10 (11)	C11—C10—C9	119.7 (4)
O4—Cd1—C8	27.19 (10)	C11—C10—H10	120.1
O5—Cd1—O1	87.40 (10)	C10—C11—Cl2	119.2 (3)
O5—Cd1—O3	97.07 (11)	C12—C11—C10	121.6 (4)
O5—Cd1—C1	83.43 (10)	C12—C11—Cl2	119.2 (3)
O5—Cd1—C8	96.30 (10)	C11—C12—C13	118.8 (4)
O6—Cd1—O1	84.21 (11)	C11—C12—H12	120.6
O6—Cd1—O2	97.43 (11)	C13—C12—H12	120.6
O6—Cd1—O3	90.46 (11)	C12—C13—H13	119.8
O6—Cd1—O4	95.42 (11)	C14—C13—C12	120.4 (4)
O6—Cd1—O5	170.03 (11)	C14—C13—H13	119.8
O6—Cd1—C1	91.39 (11)	C9—C14—H14	119.8
O6—Cd1—C8	93.37 (11)	C13—C14—C9	120.4 (4)
N1—Cd1—O1	86.30 (10)	C13—C14—H14	119.8
N1—Cd1—O2	136.21 (10)	N1—C15—C16	122.6 (3)
N1—Cd1—O3	86.21 (10)	N1—C15—H15	118.7
N1—Cd1—O4	139.00 (10)	C16—C15—H15	118.7
N1—Cd1—O5	78.90 (10)	C15—C16—C20^i^	123.4 (3)
N1—Cd1—O6	95.16 (10)	C17—C16—C15	118.4 (3)
N1—Cd1—C1	111.11 (11)	C17—C16—C20^i^	118.1 (3)
N1—Cd1—C8	112.67 (10)	C16—C17—H17	120.4
C8—Cd1—C1	135.29 (12)	C18—C17—C16	119.1 (3)
C1—O1—Cd1	88.1 (2)	C18—C17—H17	120.4
C1—O2—Cd1	95.6 (2)	C17—C18—C19	118.7 (4)
C8—O3—Cd1	90.6 (2)	C17—C18—H18	120.7
C8—O4—Cd1	93.2 (2)	C19—C18—H18	120.7
C20—O5—Cd1	124.3 (2)	N1—C19—C18	122.9 (4)
Cd1—O6—H61	107 (4)	N1—C19—H19	118.5
Cd1—O6—H62	103 (5)	C18—C19—H19	118.5
H61—O6—H62	107 (4)	O5—C20—N2	122.2 (4)
C15—N1—Cd1	122.3 (2)	O5—C20—C16^ii^	118.0 (3)
C15—N1—C19	118.2 (3)	N2—C20—C16^ii^	119.8 (3)
C19—N1—Cd1	119.0 (2)	N2—C21—C22	112.6 (4)
C20—N2—C21	125.2 (3)	N2—C21—H21A	109.1
C20—N2—C23	118.0 (4)	N2—C21—H21B	109.1
C21—N2—C23	116.5 (3)	C22—C21—H21A	109.1
O1—C1—Cd1	65.4 (2)	C22—C21—H21B	109.1
O1—C1—O2	122.5 (3)	H21A—C21—H21B	107.8
O1—C1—C2	119.7 (4)	C21—C22—H22A	109.5
O2—C1—Cd1	57.16 (19)	C21—C22—H22B	109.5
O2—C1—C2	117.7 (4)	C21—C22—H22C	109.5
C2—C1—Cd1	173.1 (3)	H22A—C22—H22B	109.5
C3—C2—C1	119.7 (3)	H22A—C22—H22C	109.5
C7—C2—C1	120.6 (4)	H22B—C22—H22C	109.5
C7—C2—C3	119.7 (3)	N2—C23—C24	112.3 (4)
C2—C3—H3	120.3	N2—C23—H23A	109.2
C4—C3—C2	119.5 (4)	N2—C23—H23B	109.2
C4—C3—H3	120.3	C24—C23—H23A	109.2
C3—C4—Cl1	120.2 (3)	C24—C23—H23B	109.2
C5—C4—Cl1	118.6 (3)	H23A—C23—H23B	107.9
C5—C4—C3	121.2 (4)	C23—C24—H24A	109.5
C4—C5—H5	120.5	C23—C24—H24B	109.5
C6—C5—C4	119.0 (4)	C23—C24—H24C	109.5
C6—C5—H5	120.5	H24A—C24—H24B	109.5
C5—C6—C7	120.5 (4)	H24A—C24—H24C	109.5
C5—C6—H6	119.8	H24B—C24—H24C	109.5
			
O2—Cd1—O1—C1	1.3 (2)	Cd1—O5—C20—N2	−109.6 (3)
O4—Cd1—O1—C1	13.0 (3)	Cd1—O5—C20—C16^ii^	72.2 (4)
O5—Cd1—O1—C1	−80.5 (2)	C15—N1—Cd1—O1	−9.4 (3)
O6—Cd1—O1—C1	104.9 (2)	C15—N1—Cd1—O2	−31.9 (3)
N1—Cd1—O1—C1	−159.5 (2)	C15—N1—Cd1—O3	164.6 (3)
C8—Cd1—O1—C1	21.4 (5)	C15—N1—Cd1—O4	178.8 (2)
O1—Cd1—O2—C1	−1.3 (2)	C15—N1—Cd1—O5	−97.5 (3)
O3—Cd1—O2—C1	−176.7 (2)	C15—N1—Cd1—O6	74.4 (3)
O4—Cd1—O2—C1	−172.8 (2)	C15—N1—Cd1—C1	−19.0 (3)
O5—Cd1—O2—C1	91.5 (2)	C15—N1—Cd1—C8	170.3 (3)
O6—Cd1—O2—C1	−78.4 (2)	C19—N1—Cd1—O1	163.1 (3)
N1—Cd1—O2—C1	27.0 (3)	C19—N1—Cd1—O2	140.6 (3)
C8—Cd1—O2—C1	−174.5 (2)	C19—N1—Cd1—O3	−22.9 (3)
O2—Cd1—O3—C8	4.5 (3)	C19—N1—Cd1—O4	−8.6 (4)
O4—Cd1—O3—C8	−0.2 (2)	C19—N1—Cd1—O5	75.1 (3)
O5—Cd1—O3—C8	90.0 (2)	C19—N1—Cd1—O6	−113.0 (3)
O6—Cd1—O3—C8	−96.5 (2)	C19—N1—Cd1—C1	153.5 (3)
N1—Cd1—O3—C8	168.3 (2)	C19—N1—Cd1—C8	−17.2 (3)
C1—Cd1—O3—C8	−0.5 (5)	Cd1—N1—C15—C16	173.3 (3)
O1—Cd1—O4—C8	174.0 (2)	C19—N1—C15—C16	0.7 (5)
O2—Cd1—O4—C8	−176.5 (2)	Cd1—N1—C19—C18	−174.4 (3)
O3—Cd1—O4—C8	0.2 (2)	C15—N1—C19—C18	−1.5 (6)
O5—Cd1—O4—C8	−95.5 (2)	C20—N2—C21—C22	−109.8 (5)
O6—Cd1—O4—C8	86.8 (2)	C23—N2—C21—C22	76.9 (5)
N1—Cd1—O4—C8	−17.5 (3)	C20—N2—C23—C24	−89.3 (5)
C1—Cd1—O4—C8	−179.9 (2)	C21—N2—C23—C24	84.5 (6)
O1—Cd1—C1—O2	177.7 (4)	O1—C1—O2—Cd1	2.4 (4)
O2—Cd1—C1—O1	−177.7 (4)	C2—C1—O2—Cd1	−174.6 (3)
O3—Cd1—C1—O1	−170.0 (3)	C3—C2—C1—O1	1.6 (5)
O3—Cd1—C1—O2	7.8 (5)	C3—C2—C1—O2	178.7 (3)
O4—Cd1—C1—O1	−170.3 (2)	C7—C2—C1—O1	−176.8 (4)
O4—Cd1—C1—O2	7.5 (2)	C7—C2—C1—O2	0.4 (5)
O5—Cd1—C1—O1	97.4 (2)	C1—C2—C3—C4	−177.5 (3)
O5—Cd1—C1—O2	−84.9 (2)	C7—C2—C3—C4	0.9 (6)
O6—Cd1—C1—O1	−74.1 (2)	C1—C2—C7—C6	177.9 (4)
O6—Cd1—C1—O2	103.6 (2)	C3—C2—C7—C6	−0.5 (6)
N1—Cd1—C1—O1	22.0 (3)	C2—C3—C4—Cl1	178.4 (3)
N1—Cd1—C1—O2	−160.3 (2)	C2—C3—C4—C5	−0.8 (6)
C8—Cd1—C1—O1	−170.3 (2)	Cl1—C4—C5—C6	−178.9 (4)
C8—Cd1—C1—O2	7.4 (3)	C3—C4—C5—C6	0.3 (7)
O1—Cd1—C8—O3	166.4 (3)	C4—C5—C6—C7	0.1 (7)
O1—Cd1—C8—O4	−13.3 (5)	C2—C7—C6—C5	0.0 (7)
O2—Cd1—C8—O3	−176.6 (2)	C10—C9—C8—O3	−11.4 (5)
O2—Cd1—C8—O4	3.7 (2)	C10—C9—C8—O4	170.1 (3)
O3—Cd1—C8—O4	−179.7 (4)	C14—C9—C8—O3	166.1 (4)
O4—Cd1—C8—O3	179.7 (4)	C14—C9—C8—O4	−12.4 (5)
O5—Cd1—C8—O3	−93.2 (2)	C8—C9—C10—C11	177.2 (3)
O5—Cd1—C8—O4	87.1 (2)	C14—C9—C10—C11	−0.3 (5)
O6—Cd1—C8—O3	84.4 (2)	C8—C9—C14—C13	−175.9 (4)
O6—Cd1—C8—O4	−95.3 (2)	C10—C9—C14—C13	1.6 (6)
N1—Cd1—C8—O3	−12.6 (3)	C9—C10—C11—Cl2	−179.9 (3)
N1—Cd1—C8—O4	167.7 (2)	C9—C10—C11—C12	−1.1 (6)
C1—Cd1—C8—O3	179.8 (2)	C10—C11—C12—C13	1.1 (7)
C1—Cd1—C8—O4	0.1 (3)	Cl2—C11—C12—C13	−180.0 (4)
Cd1—O1—C1—O2	−2.3 (4)	C11—C12—C13—C14	0.2 (7)
Cd1—O1—C1—C2	174.8 (3)	C9—C14—C13—C12	−1.6 (7)
Cd1—O3—C8—O4	0.3 (4)	N1—C15—C16—C17	1.3 (5)
Cd1—O3—C8—C9	−178.2 (3)	N1—C15—C16—C20^i^	176.9 (3)
Cd1—O4—C8—O3	−0.3 (4)	C15—C16—C17—C18	−2.4 (6)
Cd1—O4—C8—C9	178.1 (3)	C20^i^—C16—C17—C18	−178.4 (4)
C20—O5—Cd1—O1	91.2 (3)	C16—C17—C18—C19	1.7 (6)
C20—O5—Cd1—O2	37.5 (3)	N1—C19—C18—C17	0.3 (7)
C20—O5—Cd1—O3	−97.3 (3)	O5—C20—N2—C21	−173.2 (4)
C20—O5—Cd1—O4	−42.9 (3)	O5—C20—N2—C23	0.0 (5)
C20—O5—Cd1—N1	178.0 (3)	C16^ii^—C20—N2—C21	5.0 (5)
C20—O5—Cd1—C1	64.9 (3)	C16^ii^—C20—N2—C23	178.2 (3)
C20—O5—Cd1—C8	−70.1 (3)		

Symmetry codes: (i) *x*, *y*−1, *z*; (ii) *x*, *y*+1, *z*.

### Hydrogen-bond geometry (Å, º)

**Table:**

*D*—H···*A*	*D*—H	H···*A*	*D*···*A*	*D*—H···*A*
O6—H61···O2^iii^	0.85 (4)	1.94 (4)	2.753 (5)	160 (4)
O6—H62···O4^iii^	0.86 (4)	2.11 (4)	2.838 (4)	142 (5)
C15—H15···O1	0.93	2.52	3.181 (5)	128
C19—H19···O3	0.93	2.47	3.130 (5)	128

Symmetry code: (iii) −*x*+1, −*y*+2, −*z*.
